# Infant-gut associated *Bifidobacterium dentium* strains utilize the galactose moiety and release lacto-*N*-triose from the human milk oligosaccharides lacto-*N*-tetraose and lacto-*N*-neotetraose

**DOI:** 10.1038/s41598-021-02741-x

**Published:** 2021-12-02

**Authors:** Eva M. Moya-Gonzálvez, Antonio Rubio-del-Campo, Jesús Rodríguez-Díaz, María J. Yebra

**Affiliations:** 1 Laboratorio de Bacterias Lácticas y Probióticos, Departamento de Biotecnología de Alimentos, Instituto de Agroquímica y Tecnología de Alimentos, Consejo Superior de Investigaciones Científicas (IATA-CSIC), Valencia, Spain; 2 Departamento de Microbiología, Facultad de Medicina, Universidad de Valencia, Valencia, Spain

**Keywords:** Microbiology, Molecular biology

## Abstract

Much evidence suggests a role for human milk oligosaccharides (HMOs) in establishing the infant microbiota in the large intestine, but the response of particular bacteria to individual HMOs is not well known. Here twelve bacterial strains belonging to the genera *Bifidobacterium*, *Enterococcus*, *Limosilactobacillus*, *Lactobacillus*, *Lacticaseibacillus*, *Staphylococcus* and *Streptococcus* were isolated from infant faeces and their growth was analyzed in the presence of the major HMOs, 2′-fucosyllactose (2′FL), 3-fucosyllactose (3FL), 2′,3-difucosyllactose (DFL), lacto-*N*-tetraose (LNT) and lacto-*N*-*neo*-tetraose (LNnT), present in human milk. Only the isolated *Bifidobacterium* strains demonstrated the capability to utilize these HMOs as carbon sources. *Bifidobacterium infantis* Y538 efficiently consumed all tested HMOs. Contrarily, *Bifidobacterium dentium* strains Y510 and Y521 just metabolized LNT and LNnT. Both tetra-saccharides are hydrolyzed into galactose and lacto-*N*-triose (LNTII) by *B. dentium*. Interestingly, this species consumed only the galactose moiety during growth on LNT or LNnT, and excreted the LNTII moiety. Two β-galactosidases were characterized from *B. dentium* Y510, Bdg42A showed the highest activity towards LNT, hydrolyzing it into galactose and LNTII, and Bdg2A towards lactose, degrading efficiently also 6′-galactopyranosyl-*N*-acetylglucosamine, *N*-acetyl-lactosamine and LNnT. The work presented here supports the hypothesis that HMOs are mainly metabolized by *Bifidobacterium* species in the infant gut.

## Introduction

Human milk oligosaccharides (HMOs) are non-conjugated and structurally diverse carbohydrates present in human milk, with more than 100 structures already elucidated^1^. They constitute the third solid component most abundant in human milk after lactose and lipids. Concentration ranges from 10 to 20 g/l in mature milk and over 20 g/l in colostrum^2^, although relative proportions and total amounts of HMOs vary depending on the genetic background (Secretor and Lewis-based blood group) of the mother and the lactating stage^3,4^. HMOs consist of combinations of five monosaccharides: d-glucose (Glc), d-galactose (Gal), *N*-acetylglucosamine (GlcNAc), l-fucose (Fuc) and sialic acid. Within the group of neutral HMOs, 2′-fucosyllactose (2′FL; Fucα1-2Galβ1-4Glc), 3-fucosyllactose (3FL; Galβ1–4(Fucα1–3)Glc), 2′,3-difucosyllactose (DFL or LDFT; Fucα1-2Galβ1–4(Fucα1–3)Glc), lacto-*N*-tetraose (LNT; Galβ1-3GlcNAcβ1-3Galβ1-4Glc) and lacto-*N*-*neo*-tetraose (LNnT; Galβ1-4GlcNAcβ1-3Galβ1-4Glc) are the major HMOs detected in milk. LNT and LNnT constitute the core type-1 and type-2 structures, respectively, which can be further elongated by the addition of Gal, GlcNAc, Fuc or sialic acid.

HMOs are not metabolized in the gut^5^ and therefore they do not provide any nutritive value for the infant. Much evidence suggests that they have beneficial functions in infants, including an effect as immuno-modulators^6^, a decrease in the incidence of necrotizing enterocolitis in preterm infants^7^ and protection against infectious diseases through anti-adhesive antimicrobial properties^8^. Some evidences also support an important function for HMOs in building the composition of the infant gut microbiota^9,10^. Bacteria have adapted to the infant gastrointestinal tract by developing specific glycosyl hydrolases to break down HMOs^11^. Among bifidobacterial species, *Bifidobacterium longum* subsp. *infantis*, *Bifidobacterium bifidum* and *Bifidobacterium breve* readily utilize HMOs^10,12–15^. They have been shown to possess a great battery of glycosyl hydrolases involved in the catabolism of specific HMOs^10,15–18^. Other *Bifidobacterium* species such as *Bifidobacterium dentium* have also been found in infant faeces^19–21^, and it has been described that members of this species are unable to grow on HMOs^21,22^. Our group and others have demonstrated that the species of *Lacticaseibacillus casei* (previously known as *Lactobacillus casei*) and *Lactobacillus acidophillus* are also consumers of HMOs and HMO-derived glycans^23–25^, and the catabolic enzymes involved in their metabolism have been characterized^26–29^. For *Enterococcus faecalis* and *Streptococcus thermophillus* species, a slight growth was shown in fucosylated HMOs^30^.

Besides HMOs, human milk provides microorganisms that readily colonize the infant gastrointestinal tract^31^. In the present study, bacteria were isolated from breastfed infant faeces and their capability to utilize major individual HMOs (2′FL, 3FL, DFL, LNT and LNnT), HMO-derived glycans and monosaccharides for growth was determined. Among the isolated species, the *Bifidobacterium infantis* strain Y538 was found to be the most efficient at consuming all tested HMOs, and the *Bifidobacterium dentium* strains Y510 and Y521 were able to ferment the galactose moiety of LNT and LNnT. Furthermore, two β-galactosidases with activity in these tetra-saccharides were characterized from *B. dentium*.

## Results

### Isolation and identification of bacteria from breast-fed infant feces

A fecal sample mix of four exclusively breastfed infants was plated in different agar media as described in “Methods” in order to isolate bacteria. Isolates were randomly selected, subjected to RAPD-PCR analysis and at least one representative of each band pattern was kept for subsequently species identification. Based on partial 16S rRNA gene sequencing, the isolates were identified as *Bifidobacterium dentium* (Y510, Y521, Y522, Y525, Y546), *Bifidobacterium longum* (Y538), *Enterococcus faecalis* (Y513, Y515-516, Y530-534, Y536, Y547-548, Y550), *Limosilactobacillus fermentum* (ex-*Lactobacillus fermentum*) (Y500, Y502, Y504, Y506-509, Y512, Y523-524, Y527-528, Y535, Y537), *Lactobacillus gasseri* (Y511), *Lacticaseibacillus paracasei* (ex-*Lactobacillus paracasei*) (Y526), *Limosilactobacillus reuteri* (ex-*Lactobacillus reuteri*) (Y501, Y503, Y505), *Staphylococcus epidermidis* (Y520), *Staphylococcus hominis* (Y549) and *Streptococcus pasteurianus* (Y529). Previous studies have shown that RAPD-PCR analysis is an appropriate molecular tool to differentiate lactic acid bacteria at the strain level^32,33^. The RAPD-PCR profiles using the MVC primer (Supplementary Figure S1) allowed differentiating between *B. dentium* Y510 and the rest of *B. dentium* isolates. Among the *E. faecalis* isolates, all except one (Y533), showed similar band patterns. All *L. fermentum* and *L. reuteri* isolates showed a unique band pattern, respectively. One isolate representative of each band pattern was utilized for further analysis. These isolates were confirmed at the species level by sequencing the almost entire region of 16S rRNA gene. In addition, *B. dentium* isolates Y510 and Y521 were differentiated at the strain level with two more RAPD primers, PER1 and CORR1 (Supplementary Figure S2).

The two subspecies of *B. longum* that often colonize the infant intestine are *B. longum* subsp. *infantis* and *B. longum* subsp. *longum*. Based on the restriction patterns of the partial 16S rRNA gene amplicon digested with Sau3AI^34^, the *B. longum* Y538 strain was consistent with *B. longum* subsp. *infantis* (data not shown).

### Growth of bacteria isolated strains on individual HMOs

**Figure 1:**
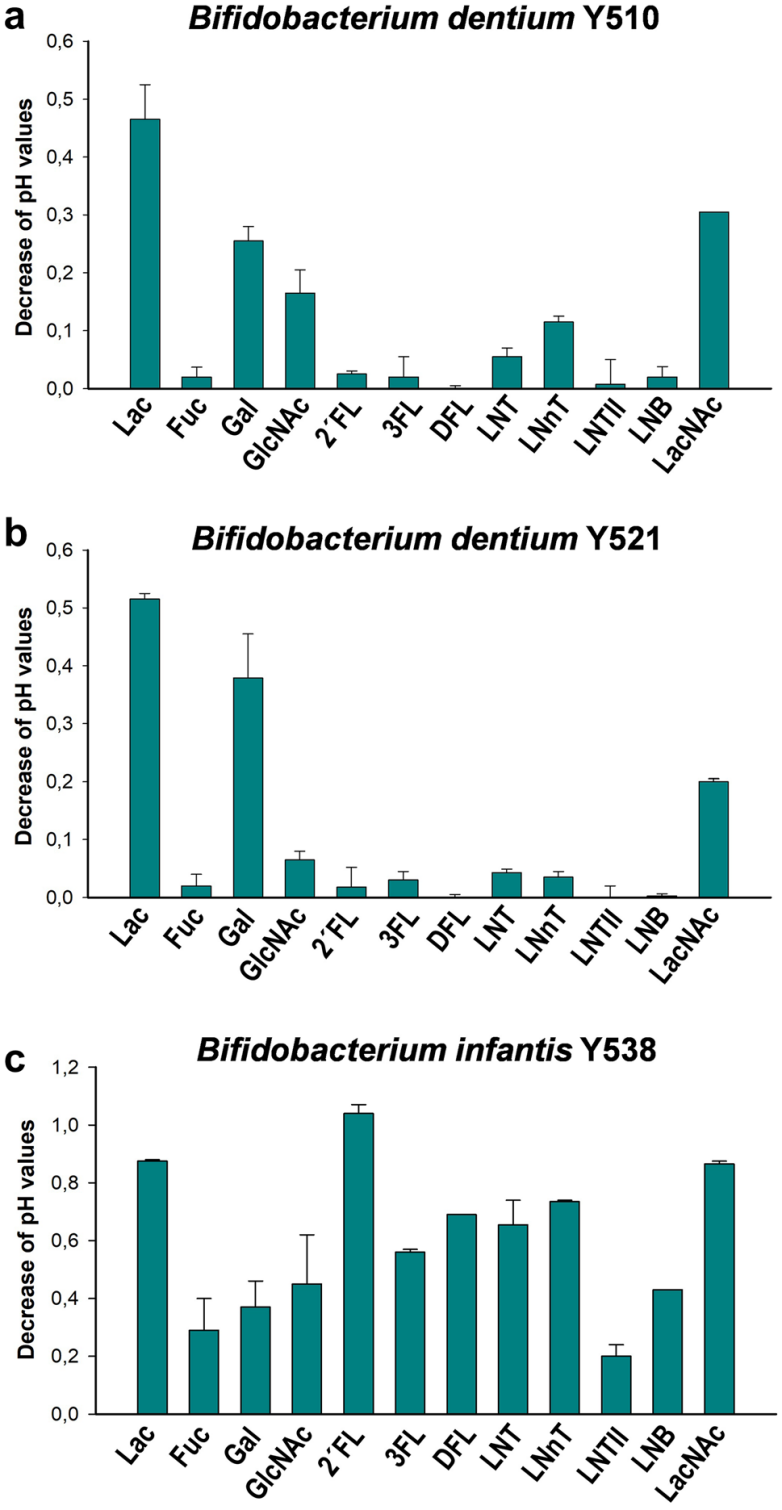
Carbohydrate utilization profiles of *Bifidobacterium dentium* Y510 (**a**), *B. dentium* Y521 (**b**) and *Bifidobacterium infantis* Y538 (**c**). MRS basal medium supplemented with 2 mM of carbohydrates was used. pH decrease values of culture supernatants are shown. Data presented are mean values based on at least two replicates. Errors bars indicated standard deviations. *Lac *lactose, *Fuc* l-fucose, *Gal* galactose, *GlcNAc* *N*-acetylglucosamine, *2′FL* 2*′*-fucosyllactose, *3FL* 3-fucosyllactose, *DFL* difucosyllactose, *LNT* lacto-*N*-tetraose, *LNnT* lacto-*N*-neotetraose, *LNTII* lacto-*N*-triose, *LNB* lacto-*N*-biose, *LacNAc* *N*-acetyllactosamine.

In order to determine the ability of the newly obtained bacterial strains to utilize HMOs as a carbon source, their ability to grow on MRS basal medium supplement with 2′-fucosyllactose (2′FL), 3-fucosyllactose (3FL), 2′,3-difucosyllactose (DFL), lacto-*N*-tetraose (LNT) or lacto-*N*-neotetraose (LNnT) was analyzed. From the twelve bacteria strains assayed, only *B. dentium* and *B. infantis* strains were able to grow in the presence of HMOs (Table 1). *B. dentium* Y510 and Y521 strains utilized LNT and LNnT, although with a low efficiency as seen by the modest reduction in pH in the culture medium (Fig. 1). *B. infantis* Y538 grew in the presence of all five oligosaccharides (Fig. 1). The strains *E. faecalis* (Y513, Y533), *L. fermentum* (Y500), *L. gasseri* (Y511), *L. paracasei* (Y526), *L. reuteri* (Y501), *S. epidermidis* (Y520), *S. hominis* (Y549) and *S. pasteurianus* (Y529) did not exhibit any significant growth in the presence of the tested HMOs compared to the culture controls without carbohydrate source (Table 1; Supplementary Figure S3). The growth of the isolates in the presence of lactose or GlcNAc as positive controls is also shown. All bacterial strains, except *E. faecalis* Y533 and *S. hominis* Y549, were able to grow in the presence of lactose, and these two strains utilized GlcNAc. The fermentation of l-fucose was also assayed and the *B. infantis* and *S. pasteurianus* tested strains were able to catabolize this monosaccharide (Table 1).

**Table 1:** Utilization of monosaccharides and human milk oligosaccharides by infant-gut associated bacterial strains^a^.

Strains	Lac	Fuc	Gal	GlcNAc	2*′*FL	3FL	DFL	LNT	LNnT	LNTII	LNB	LacNAc
*B. dentium* Y510	++	−	++	+	−	−	−	±	+	−	−	+
*B. dentium* Y521	++	−	++	±	−	−	−	±	±	−	−	+
*B. infantis* Y538	+++	+	++	++	+++	+++	+++	+++	+++	+	++	+++
*E. faecalis* Y513	+++	−	nd	++	−	−	−	−	−	nd	nd	nd
*E. faecalis* Y533	−	−	nd	+++	−	−	−	−	−	nd	nd	nd
*L. fermentum* Y500	+++	−	nd	−	−	−	−	−	−	nd	nd	nd
*L. gasseri* Y511	++	−	nd	+	−	−	−	−	−	nd	nd	nd
*L. paracasei* Y526	+++	−	nd	++	−	−	−	−	−	nd	nd	nd
*L. reuteri* Y501	+++	−	nd	−	−	−	−	−	−	nd	nd	nd
*S. epidermidis* Y520	+++	−	nd	−	−	−	−	−	−	nd	nd	nd
*S. hominis* Y549	−	−	nd	+++	−	−	−	−	−	nd	nd	nd
*S. pasteurianus* Y529	++	+	nd	++	−	−	−	−	−	nd	nd	nd

^a^Level of bacterial growth was classified as follows: *Bifidobacterium* strains, OD_595_ < 0.12 (−), OD_595_ 0.12–0.2 (±), OD_595_ 0.2–0.3 (+), OD_595_ 0.3–0.5 (++) and OD_595_ > 0.5 (+++); *E. faecalis* Y513, OD_595_ < 1.0 (−), OD_595_ 1.0–1.2 (++) and OD_595_ > 1.2 (+++); *E. faecalis* Y533, OD_595_ < 0.5 (−) and OD_595_ > 0.5 (+++); *L. fermentum*, OD_595_ < 0.6 (−) and OD_595_ > 0.6 (+++); *L. gasseri* and *S. pasteurianus*, OD_595_ < 0.2 (−), OD_595_ 0.2–0.3 (+) and OD_595_ > 0.3 (++); *L. paracasei* and *L. reuteri*, OD_595_ < 0.35 (−), OD_595_ 0.35–0.45 (++) and OD_595_ > 0.45 (+++); *S. epidermidis*, OD_595_ < 5.0 (−) and OD_595_ > 5.0 (+++); *S. hominis*, OD_595_ < 3.0 (−) and OD_595_ > 3.0 (+++). OD_595_ values correspond to 48 h cultures (*Bifidobacterium* strains) and maximum OD values for the rest of the strains.

*nd* not determined.

*Lac* lactose, *Fuc* L-fucose, *Gal* galactose, *GlcNAc* *N*-acetylglucosamine, *2′FL* 2*′*-fucosyllactose, *3FL* 3-fucosyllactose, *DFL* difucosyllactose, *LNT* lacto-*N*-tetraose, *LNnT* lacto-*N*-neotetraose, *LNTII* lacto-*N*-triose, *LNB* lacto-*N*-biose, *LacNAc* *N*-acetyllactosamine.

**Figure 2:**
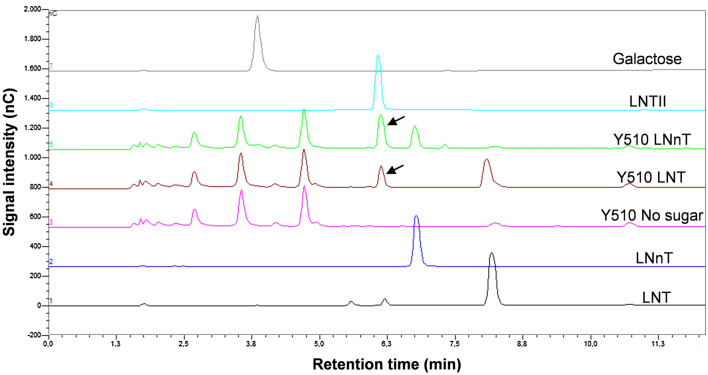
HPLC chromatograms (Dionex system) of the standard compounds LNT (chromatogram 1), LNnT (chromatogram 2), LNTII (chromatogram 6) and galactose (chromatogram 7), and culture supernatants from *Bifidobacterium dentium* Y510 grown on MRS basal medium without sugar (chromatogram 3), with LNT (chromatogram 4) or LNnT (chromatogram 5). The arrows showed the LNTII present in the culture supernatants. *LNT* lacto-*N*-tetraose, *LNnT* lacto-*N*-neotetraose, *LNTII* lacto-*N*-triose, *nC* nanoCoulomb.

To confirm that the HMOs were or not fermented by the isolated strains, the supernatants of the cultures were analyzed for sugar content. No measurable decrease in HMOs concentration was observed for the isolates, with the exception of the *Bifidobacterium* species. *B. dentium* strains partially degraded LNT and LNnT (Table 2). Unexpectedly, lacto-*N*-triose (LNTII) was detected in the culture supernatant of both strains when grown in either tetra-saccharide. In Fig. 2 are presented the carbohydrate analysis of the culture supernatants from Y510 strain. The amount of LNTII present in the supernatants corresponded to the amount of LNT or LNnT consumed, respectively, indicating that that intermediate degradation compound is not metabolized. To further confirm that the LNTII was not fermented, the growth of Y510 and Y521 strains was analyzed in MRS medium supplement with LNTII. The results showed that both strains failed to growth on LNTII and this tri-saccharide remained in the spent supernatants (Fig. 1; Table 2). Galactose was not accumulated in the supernatants of *B. dentium* when grown in LNT or LNnT (Fig. 2) and that monosaccharide is substrate for this species (Table 1, Fig. 1). These results indicate that the growth supported by LNT or LNnT is only due to the metabolism of the galactose moiety. The *B. infantis* Y538 strain completely depletes all tested HMOs from the culture medium (Table 2) and no intermediate degradation components were observed. The *Bifidobacterium* strains were also tested for the ability to utilize the disaccharides lacto-*N*-biose (LNB) and *N*-acetyllactosamine (LacNAc), which form part of the LNT and LNnT molecules, respectively, as carbon sources. The results showed that both *B. dentium* strains metabolize LacNAc but not LNB, and *B. infantis* Y538 consumed both carbohydrates (Fig. 1, Table 2).

**Table 2:** Percentage of oligosaccharide utilized by the *Bifidobacterium* strains in the fermentation conditions described in “Methods”.

Strains	2*′*FL	3FL	DFL	LNT	LNnT	LNTII	LNB	LacNAc
*B. dentium* Y510	0.0	0.0	0.0	19.0 ± 6.0	80.5 ± 5.5	0.0	0.0	88.5 ± 0.5
*B. dentium* Y521	0.0	0.0	0.0	9.0 ± 0.0	12.5 ± 0.5	0.0	0.0	33.0 ± 1.0
*B. infantis* Y538	98.0 ± 2.0	100	99.0 ± 1.0	100	100	24.0 ± 2.0	100	100

*2′FL* 2*′*-Fucosyllactose, *3FL* 3-fucosyllactose, *DFL* difucosyllactose, *LNT* lacto-*N*-tetraose, *LNnT* lacto-*N*-neotetraose, *LNTII* lacto-*N*-triose, *LNB* lacto-*N*-biose, *LacNAc* *N*-acetyllactosamine.

### Cellular location of the β-galactosidase activity involved in LNT and LNnT hydrolysis in *B. dentium*

**Figure 3:**
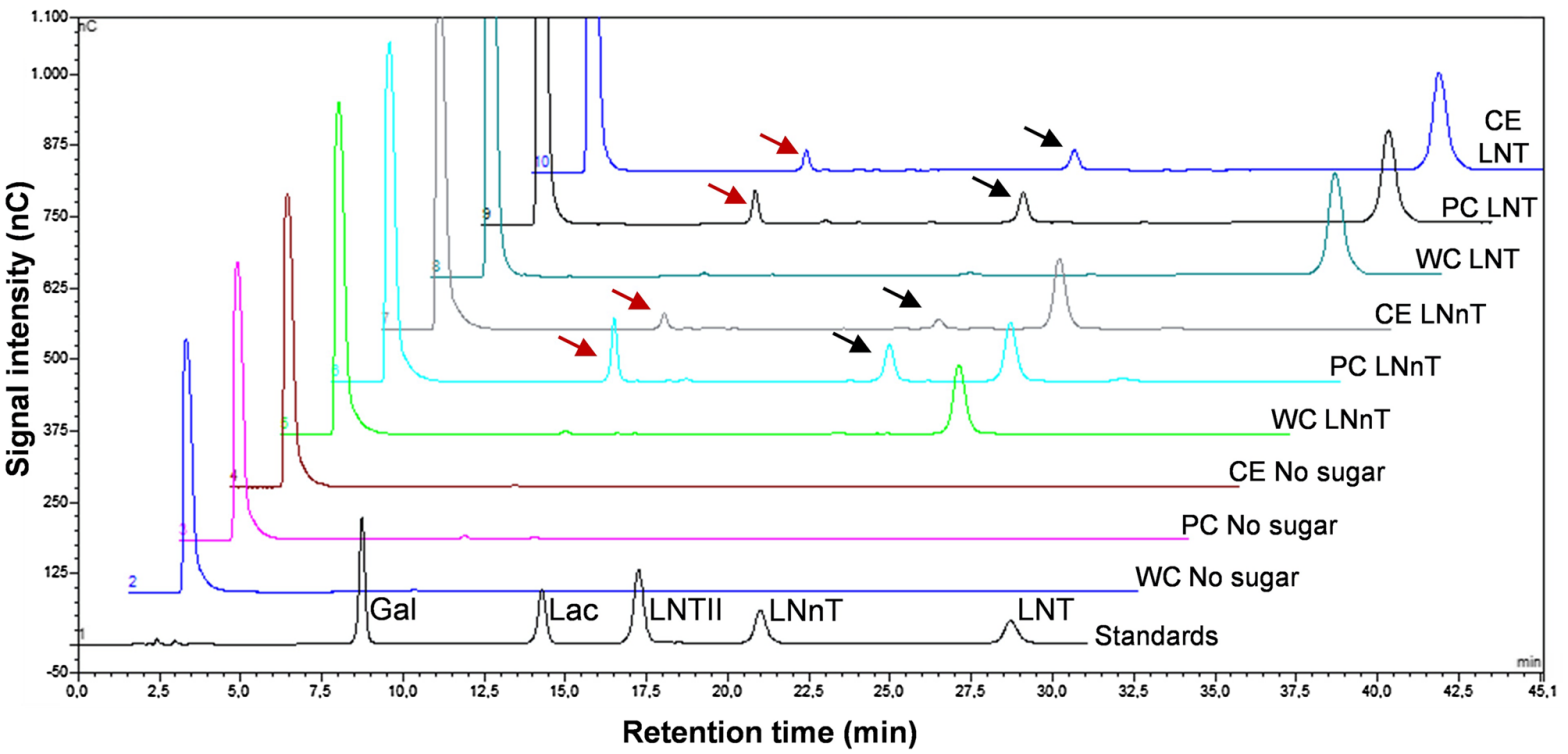
HPLC chromatograms (Dionex system) of standard compounds mixture (chromatogram 1) and reaction mixtures containing *Bifidobacterium dentium* Y510: whole cells (WC) without sugar (chromatogram 2), with LNnT (chromatogram 5) or LNT (chromatogram 8); permeabilized cells (PC) without sugar (chromatogram 3), with LNnT (chromatogram 6) or LNT (chromatogram 9) and cell-free crude extracts (CE) without sugar (chromatogram 4), with LNnT (chromatogram 7) or LNT (chromatogram 10). The red and black arrows showed the Gal and LNTII, respectively present in the reaction mixtures. *LNT* lacto-*N*-tetraose, *LNnT* lacto-*N*-neotetraose, *LNTII* lacto-*N*-triose, *Lac* lactose, *Gal* galactose, *nC* nanoCoulomb.

As shown above, during the growth of *B. dentium* in LNT or LNnT, peaks of LNTII were detected in the culture medium. However, no β-galactosidase activity was detected in the cell-free supernatant, indicating that the enzymes responsible of the hydrolysis of these HMOs are physically associated with cells (Supplementary Table S1). In order to determine their localization in the cells, the hydrolysis of LNT and LNnT, respectively, was analyzed using whole cells, permeabilized cells and cell-free crude extracts of *B. dentium* strain Y510 (Fig. 3). LNT and LNnT are broken down to LNTII and galactose by the permeabilized cells and cell-free crude extracts but not by the whole cells. These results indicate that the β-galactosidases responsible of the LNT and LNnT hydrolysis in *B. dentium* are intracellular.

### Galactosidases from *B. dentium* hydrolyze LNT and LNnT

The β-galactosidases Bga42A and Bga2A release β-1,3 and β-1,4 linked galactose from LNT and LNnT, respectively, in *B. infantis*^18^. A BLAST search using the deduced amino acid sequence of Bga42A or Bga2A against the genome of *B. dentium* JCM 1195 type strain shows the highest homology against locus BBDE_RS03200 (75% identities; 85% positives) and BBDE_RS07935 (57% identities; 72% positives), respectively. These genes encode putative β-galactosidases of the GH42 and GH2 glycoside hydrolase families (http://www.cazy.org), and sequence analysis using the SignalP (version 5.0) program (http://www.cbs.dtu.dk) showed that they do not have *N*-terminal signal peptides, suggesting that they are intracellular enzymes. Based on this, specific primers were used to search for genes homologues to BBDE_RS03200 and BBDE_RS07935 in the *B. dentium* Y510 isolate. The results showed that this strain contains genes homologues to those. In order to investigate the predicted enzymatic activities encoded by BBDE_RS03200 homolog (designated as *bdg42A*) and BBDE_RS07935 homolog (designated as *bdg2A*) on LNT and LNnT, those genes were cloned in *Escherichia coli* and the corresponding proteins Bdg42A and Bdg2A were purified as His-tagged fusions. They showed a molecular mass of 79 and 118 kDa, respectively, in agreement with the estimated mass of the 6xHis-tagged Bdg42A (79,112 Da) and 6xHis-tagged Bdg2A (118,010 Da) (Supplementary Figure S4). To determine their substrate specificities, both enzymes were first tested for hydrolysis of different 2/4-nitrophenyl (NP) sugars and they only showed activity on 2/4-NP-β-d-galactopyranosides, thus confirming their β-galactosidase specificity (Tables 3, 4). The general properties of Bdg42A and Bdg2A were determined using the substrate 4-NP-β-d-galactopyranoside as follows, respectively: V_max_ (μmol/min/mg protein), 25.0 and 14.3; K_m_ (mM), 2.5 and 10.0; optimum pH, pH 7.0 and pH 7.5; optimum temperature, 40 °C and 42 °C.

**Table 3:** Activity of enzyme Bdg42A.

Substrate^a^ (structure)	Hydrolysis^b^	*K*_m_ (mM)	*k*_cat_ (s^-1^)	*k*_cat_/*K*_m_ (mM^-1^ s^-1^)
2-NP-β-d-galactopyranoside	+			
4-NP-β-d-galactopyranoside	+			
4-NP-β-d-glucopyranoside	−			
2-NP-1-thio- β-d-galactopyranoside	−			
4-NP-β-d-glucuronide	−			
4-NP-*N*-acetyl-β-d-glucosaminide	−			
4-NP-α-d-glucopyranoside	−			
4-NP-α-l-fucopyranoside	−			
4-NP-α-d-galactopyranoside	−			
Galacto-*N*-biose (Galβ1-3GalNAc)	+	8.72	0.06	0.006
Lacto-*N*-biose (Galβ1-3GlcNAc)	+	10.05	1.85	0.184
Lacto-*N*-triose (GlcNAcβ1-3Galβ1-4Glc)	−			
Lacto-*N*-tetraose (Galβ1-3GlcNAcβ1-3Galβ1-4Glc)	+	5.60	22.20	3.964
Lacto-*N*-neotetraose (Galβ1-4GlcNAcβ1-3Galβ1-4Glc)	+	10.85	1.00	0.092
6*′*-Galactopyranosyl-GlcNAc (Galβ1-6GlcNAc)	+	7.72	0.29	0.037
3*′*-*N*-Acetylgalactosaminyl-Gal (GalNAcβ1-3Gal)	−			
3*′*-*N*-Acetylglucosaminyl-Man (GlcNAcβ1-3Man)	−			
Lactose (Galβ1-4Glc)	+	8.46	7.14	0.843
*N*-Acetyl-lactosamine (Galβ1-4GlcNAc)	+	10.45	0.02	0.002
Lactulose (Galβ1-4Fru)	+	11.25	3.63	0.322
Maltose (Glcα1-4Glc)	−			
*N*-Acetyl-chitobiose (GlcNAcβ1-4GlcNAc)	−			

^a^Carbohydrates used as substrates. *NP* nitrophenyl, *Glc* glucose, *Gal* Galactose, *GlcNAc* *N*-acetylglucosamine, *GalNAc* *N*-acetylgalactosamine, *Man*, mannose, *Fru* fructose.

^b^+, substrate is totally hydrolyzed after 16 h reaction in the conditions described in “Methods”; − , no activity detected.

**Table 4:** Activity of enzyme Bdg2A.

Substrate^a^ (structure)	Hydrolysis^b^	*K*_m_ (mM)	*k*_cat_ (s^-1^)	*k*_cat_/*K*_m_ (mM^-1^ s^-1^)
2-NP-β-d-galactopyranoside	+			
4-NP-β-d-galactopyranoside	+			
4-NP-β-d-glucopyranoside	−			
2-NP-1-thio-β-d-galactopyranoside	−			
4-NP-β-d-glucuronide	−			
4-NP-N-acetyl-β-d-glucosaminide	−			
4-NP-α-d-glucopyranoside	−			
4-NP-α-l-fucopyranoside	−			
4-NP-α-d-galactopyranoside	−			
Galacto-*N*-biose (Galβ1-3GalNAc)	+/−	nd	nd	
Lacto-*N*-biose (Galβ1-3GlcNAc)	+/−	nd	nd	
Lacto-*N*-triose (GlcNAcβ1-3Galβ1-4Glc)	−			
Lacto-*N*-tetraose (Galβ1-3GlcNAcβ1-3Galβ1-4Glc)	+/−	nd	nd	
Lacto-*N*-neotetraose (Galβ1-4GlcNAcβ1-3Galβ1-4Glc)	+	6.75	0.67	0.099
6*′*-Galactopyranosyl-GlcNAc (Galβ1-6GlcNAc)	+	6.99	0.083	0.012
3*′*-*N*-Acetylgalactosaminyl-Gal (GalNAcβ1-3Gal)	−			
3*′*-*N*-Acetylglucosaminyl-Man (GlcNAcβ1-3Man)	−			
Lactose (Galβ1-4Glc)	+	5.59	1.21	0.216
*N*-Acetyl-lactosamine (Galβ1-4GlcNAc)	+	6.69	0.154	0.023
Lactulose (Galβ1-4Fru)	+/−	nd	nd	
Maltose (Glcα1-4Glc)	−			
*N*-Acetyl-chitobiose (GlcNAcβ1-4GlcNAc)	−			

*nd* not determined.

^a^Carbohydrates used as substrates. *NP* nitrophenyl, *Glc* glucose, *Gal* Galactose, *GlcNAc* *N*-acetylglucosamine, *GalNAc* *N*-acetylgalactosamine, *Man* mannose, *Fru* fructose.

^b^+, substrate is totally hydrolyzed after 16 h reaction in the conditions described in “Methods”; +/−, substrate is partially hydrolyzed after 16 h reaction in the conditions described in “Methods”; − , no activity detected.

Among the HMOs and other natural oligosaccharides tested, Bdg42A hydrolyzed the disaccharides galacto-*N*-biose (GNB), LNB, 6′-galactopyranosyl-GlcNAc, lactose, LacNAc and lactulose (Table 3). As well, Bdg42A removed the galactose moiety at the non-reducing end of LNT and LNnT. This enzyme did not act on LNTII, 3′-*N*-acetylgalactosaminyl-Gal, 3′-*N*-acetylglucosaminyl-Man, maltose and *N*-acetyl-chitobiose. These results showed therefore that Bdg42A is an exo-β-galactosidase. The enzyme releases galactose from LNT with a high catalytic efficiency compared to that observed for the other substrates evaluated, suggesting a key role in the metabolism of this HMO (Table 3).

Regarding the substrate specificity of Bdg2A on the oligosaccharides assayed, this enzyme hydrolyzed the disaccharides GNB, LNB, 6′-galactopyranosyl-GlcNAc, lactose, LacNAc and lactulose into its monosaccharides constituents, and the tetra-saccharides LNT and LnNT into galactose and LNTII (Table 4). As described for Bdg42A, these results demonstrated that this enzyme is also an exo-β-galactosidase. Bdg2A showed the highest catalytic activity for lactose and it also hydrolyzed 6′-galactopyranosyl-GlcNAc, LacNAc and LNnT (Table 4).

## Discussion

HMOs have been proposed as the main metabolites from human milk that directly influence the microbiota composition of the infant gastrointestinal tract^9,10^. Several studies have shown that specific bifidobacterial species including *B. longum* subsp. *infantis*, *B. bifidum* and *B. breve* can grow efficiently on HMOs, and their genomes are equipped with genes coding for glycosidases linked to HMOs metabolism^10,12,14,15^. However, the consumption of individual HMOs by other bacterial species commonly present in the infant gut is not as well established. In this work, bacteria were isolated from breastfed infant faeces and their capability to consume the oligosaccharides 2′FL, 3FL, DFL, LNT and LNnT, that are present in human breast milk, was analyzed. The isolated strains belonging to the species *E. faecalis*, *L. fermentum*, *L. gasseri*, *L. paracasei*, *L. reuteri*, *S. epidermidis*, *S. hominis* and *S. pasteurianus* did not utilize any of the HMOs tested. However, *B. infantis* and *B. dentium* isolated strains metabolized those HMOs, contributing to the hypothesis that these carbohydrates mainly promote the growth of *Bifidobacterium* species in the infant gut. Importantly, the extent to which both species utilized HMOs was very different. While *B. infantis* totally consumed all tested HMOs and resulting intermediary degradation carbohydrates, *B. dentium* only degraded LNT and LNnT. The two subspecies of *B. longum* that often colonize the infant intestine are *B. longum* subsp. *infantis* and *B. longum* subsp. *longum*, and they differ in their ability to metabolize HMOs. While in the latest subspecies the capacity to metabolize HMOs is limited to specific strains and to certain types of HMOs^35^, in *B. infantis* the ability to consume a wide range of HMOs is characteristic of the entire subspecies^13,14,36^. The ability of the *B. infantis* Y538 strain to ferment all the HMOs assayed here is in agreement with the high capacity to consume HMOs widely described for *B. infantis*^13,14,36^. This species contains genes encoding α-l-fucosidases involved in the metabolism of 2′FL, 3FL and DFL^14^. Recently, two FL transporters with distinct but overlapping functions involved in the assimilation of these HMOs have also been characterized^37^. As well, β-galactosidases for LNT and LNnT have already been characterized in this species^18^.

Contrarily to *B. infantis*, the isolated strains Y510 and Y521 of *B. dentium* grow inefficiently in the presence of LNT or LNnT as carbon sources, utilizing only the galactose moiety and releasing LNTII into the environment. The accumulation of this tri-saccharide in the culture media as a result of LNnT degradation has also been demonstrated for *L. acidophillus* strain NCFM, which has an extracellular β-galactosidase active on LNnT^25^. Unlike this strain, the hydrolysis of LNT and LNnT by permeabilized cells, but not by whole cells of *B. dentium* strain Y510, indicated that the β-galactosidases act intracellularly on those carbohydrates. Previous results have shown in *L. casei* that fucosyl-oligosaccharides and fucosylated *N*-glycopeptides are hydrolyzed inside the cells by the α-l-fucosidases AlfB and AlfC, and that the released l-fucose is excreted into the environment^26,38^. The β-galactosidase Bdg42A characterized here is homologous to the β-galactosidases Bga42A (75% identities; 85% positives) and LntA (76% identities; 86% positives) previously described from *B. infantis* and *B. breve*, respectively^16,18^. The three enzymes exhibit the highest activity on LNT and hydrolyze it into galactose and LNTII. For *B. breve* species, it has been shown that LNT is intracellularly hydrolyzed by LntA^16^, but whether Bdg42A has the same role in *B. dentium*, further analyses are needed. The other β-galactosidase, Bdg2A, analyzed here showed homology to Bga2A (57% identities; 73% positives) from *B. infantis*, and to LacZ2 (62% identities; 73% positives) and LacZ6 (60% identities; 71% positives) from *B. breve*^16,18^. These four enzymes showed hydrolytic activity on type-2 oligosaccharides, including lactose and LNnT, and they are essentially inactive on type-1 oligosaccharides. These results suggest a similar function of those β-galactosidases in HMOs metabolism.

The LNTII resulting from the metabolism of LNT and LNnT in *B. infantis* and *B. breve* is further catabolized by β-*N*-acetylglucosaminidases of the GH20 glycoside hydrolase family that release GlcNAc and lactose^16,39^. Curiously, the LNTII moiety resulting from the degradation of LNT or LNnT by *B. dentium* was accumulated quantitatively in the culture supernatants. In agreement with this, only two out of 38 *B. dentium* strains analyzed contain genes predicted to encode glycosidases belonging to the GH20 family^40^. Previous studies have demonstrated that degradation of HMOs by some *Bifidobacterium* species support growth of other species by cross-feeding on liberated carbohydrates^41^. The LNTII excreted into the environment from LNT and LNnT metabolism by *B. dentium* could allow growth of other species within the gut ecosystem. Indeed, bacterial species associated with the infant gastrointestinal tract have been described to utilize LNTII as carbon source^28^.

The results obtained in the present work showed differential utilization of HMOs, HMO-derived glycans and monosaccharides among specific bacteria isolated from infant faeces. In particular *B. dentium*, while previously shown as an opportunistic pathogen in the oral cavity rich in simple sugars^42^, remained uncharacterized how this bacterium is able to adapt to the infant gut, where complex carbohydrates such as HMOs are abundant. Our findings have provided insights into the utilization of type 1 and type 2 HMOs by *B. dentium*, and the possible involvement of specific β-galactosidase enzymes in its metabolism. Future work, including whole genome sequencing of the *B. dentium* strains characterized here, would be needed to provide additional understanding of oligosaccharide metabolism by these bacteria.

## Methods

### Bacteria isolation from infant fecal samples

Stool samples from four breastfed infants between one and three months old were collected, stored and cryopreserved as previously described^43^. Serial dilutions (10^–3^–10^–8^) of a fecal sample mix were plated on Rogosa agar medium (Pronadisa), MRS basal^27^ agar medium supplemented with lactulose 0.5% or inulin 0.5%, MRS (Difco) agar medium with mupirocin 50 mg/L or nalidixic acid 25 mg/L. All MRS agar media contain also cysteine 0.1%. The culture plates were incubated in anaerobic jars at 37 °C during 48 h. One hundred and fifty colonies (50 colonies from the Rogosa agar medium and 25 colonies from each of the four different MRS agar media) were randomly selected at the lowest dilutions giving single colonies, and subjected to randomly amplified polymorphic DNA (RAPD) analysis. RAPD-PCR reactions were performed as previously described^44^ and using the primers MCV (AGTCAGCCAC)^45^, PER1 (AAGAGCCCGT)^33^ and CORR1 (TGCTCTGCCC)^33^. The reaction products were analyzed by agarose gel electrophoresis. 16S rRNA gene of representative isolates was amplified by PCR using cells from the colonies as the template and the primers 27F (AGAGTTTGATCCTGGCTCAG)^46^, 924R (CTTGTGCGGGCCCCCGTCAATTC)^47^ and 1492R (GGTTACCTTGTTACGACTT)^48^. The PCR products were sequenced by Eurofins Genomics (http://www.eurofinsgenomics.com). The sequences were used in BLAST searches to identify each isolated.

### Culture of bacterial strains with individual HMOs, HMOs-derived carbohydrates and monosaccharides

The isolated strains belonging to the genera *Enterococcus*, *Limosilactobacillus*, *Lactobacillus*, *Lacticaseibacillus*, *Staphylococcus* and *Streptococcus* were grown overnight at 37 °C under static conditions as previously described^27^ on MRS basal medium containing: bactopeptone (Difco), 10 g l^−1^; yeast extract (Pronadisa), 4 g l^−1^; sodium acetate, 5 g l^−1^; tri-ammonium citrate, 2 g l^−1^; magnesium sulphate 7-hydrate, 0.2 g l^−1^; manganese sulphate monohydrate, 0.05 g l^−1^; Tween 80, 1 ml l^−1^ and l-cysteine 0.1%. Overnight cultures were diluted to an optical density at 595 nm of 0.1 in 100 µl of MRS basal medium containing 2 mM lactose, l-fucose, *N*-acetylglucosamine (GlcNAc), 2′-fucosyllactose (2′FL), 3-fucosyllactose (3FL), 2′,3-difucosyllactose (DFL), lacto-*N*-tetraose (LNT) or lacto-*N*-neotetraose (LNnT). All the oligosaccharides were obtained from Biosynth Carbosynth (Compton, Berkshire, United Kingdom). Bacterial growth was tracked by measuring at 595 nm during 24 h at 37 °C in 96-well plates in a POLARstar Omega microplate reader (BMG Labtech, Offenburg, Germany). The growth of the isolated *Bifidobacterium* strains in the presence of lactose, l-fucose, galactose, GlcNAc, 2′FL, 3FL, DFL, LNT, LNnT, lacto-*N*-triose (LNTII), lacto-*N*-biose (LNB) or *N*-acetyllactosamine (LacNAc) at 2 mM was tested in MRS basal medium as previously described^23^ with some modifications. Briefly, the *Bifidobacterium* strains were cultured overnight on MRS basal medium and diluted to an OD_595_ of 0.1 in 200 μl of MRS basal medium supplemented with each carbohydrate in 96-well plates. Cultures were grown at 37 ºC under anaerobic conditions using an anaerobic atmosphere generation system (Anaerogen, Oxoid). Bacterial growth was determined by measuring the pH of the culture and OD (595 nm) at 48 h. At least two independent biological replicates and three technical replicates were performed for each growth assay. Results are expressed as means ± standard deviations.

### HMOs, HMOs-derived carbohydrates and monosaccharides analysis in culture supernatants

To determine the carbohydrates present in the supernatants from the isolated strains cultures, cells were removed by centrifugation and the supernatants were filtrated and analyzed by high-performance liquid chromatography using an ICS3000 chromatographic system (Dionex) and a CarboPac PA100 column with pulsed amperometric detection. A gradient of NaOH was used at 27 °C and at a flow rate of 1 ml/min for the analysis of fucosyl-oligosaccharides (10–100 mM NaOH for 15 min), and a combined gradient of NaOH and acetic acid was used at the same temperature and flow rate for the rest of the oligosaccharides analyzed (100 mM NaOH for 2 min, 100–300 mM NaOH for 3 min, 300 mM NaOH and 0–300 mM acetic acid for 15 min). Monosaccharides and oligosaccharides were confirmed by comparison of their retention times with those of standards.

### β-Galactosidase activity in supernatants, whole cells, permeabilized cells and cell-free crude extracts of *B. dentium* Y510 cultures

The *B. dentium* Y510 strain was grown overnight at 37 °C on 50 ml of MRS basal medium^27^ supplemented with 0.1% l-cysteine and 0.5% glucose, and under anaerobic conditions using an anaerobic atmosphere generation system (Anaerogen, Oxoid, Basingstoke, UK). Cells were collected by centrifugation, washed with Tris–HCl buffer 50 mM, pH 7.5 and suspended in this buffer to an OD_595_ of 2. Cells were permeabilized using deoxycholic acid as previously described with some modifications^49^. Four hundred microliters of cell suspension were incubated with 400 μl of deoxycholic acid 20 mM under agitation for 5 min. Cell-free crude extract was prepared as previously described^50^. Protein concentration in the crude extracts was determined with the Protein Assay Dye Reagent Concentrate (BioRad). The β-galactosidase enzyme activity was determined by measuring the 2-nitrophenol released (absorbance at 404 nm) from 2-nitrophenyl (NP)-β-d-galactopyranoside (oNPGal) at 37 °C in 96-well plates (POLARstar Omega microplate reader, BMG Labtech). The reaction mixtures (50 μl) containing 100 mM Tris–HCl buffer pH7.0, 5 mM oNPGal were started by adding 40 μl of culture supernatant, 10 μl of whole cells, 10 μl of permeabilized cells or 10 μl of cell-free crude extract.

The β-galactosidase activity on LNT and LNnT was determined using reaction mixtures (10 μl) containing 100 mM Tris–HCl buffer pH7.0, 5 mM LNT or LNnT, and 8.5 μl of whole cells, permeabilized cells or cell-free crude extract. The reactions were incubated at 37 °C overnight, and after been diluted 10 times they were analyzed by chromatography using the Dionex system and column described above. A gradient of 10 mM to 150 mM NaOH was used at 27 °C for 30 min at a flow rate of 1 ml/min.

### Expression and purification of His-tagged Bdg42A and Bdg2A

Total DNA was isolated from *B. dentium* Y510 using the MasterPure DNA extraction Kit (Epicentre) following the manufacturer’s protocols with some modifications^44^. The coding regions of *bdg42A* and *bdg2A* were amplified by PCR with the Phusion High-Fidelity DNA polymerase (Thermoscientific) using genomic DNA from *B. dentium* Y510 and the primers pairs: BDG3SacIF (5′-TTTTGAGCTCATGACGCAGCGCAGAGCAC)/ BDG3HindIIIR (5′-TTTTAAGCTTTTACTTCCTGAGCACGATTACG) and BDG4SacIF (5′-TTTTGAGCTCATGTCGCATATCTTTTCCTCAAC)/ BDG4HindIIIR (5′-TTTTAAGCTTTCAGAACAGCTCCAGCATCAC), respectively, to which restriction sites (underlined) were added to the 5′ and 3′ ends. The digested PCR products were cloned into pQE80 (Qiagen) and the resulting plasmids, pQEbdg42A and pQEbdg2A, respectively, were transformed by electroporation into *Escherichia coli* DH10B. *E. coli* transformants were selected with ampicillin (100 μg/ml), and DNA sequencing allowed to verified the correct sequence of the inserts. One clone of each, PE177 (pQEbdg42A) and PE178 (pQEbdg2A), was grown in Luria–Bertani medium (Oxoid), induced with IPTG (isopropyl-β-d-thiogalactopyranoside; 1 mM) and the recombinant proteins purified from the cleared extracts as described previously^27^. SDS-PAGE was used to determine the fractions containing the proteins of interest, which were kept frozen at − 80 °C with 20% glycerol. Protein concentrations were determined with the Protein Assay Dye Reagent Concentrate (BioRad).

### Bdg42A and Bdg2A enzyme activities

The activity of the purified Bdg42A and Bdg2A enzymes were assayed with 2/4-NP-sugars (Tables 3, 4) at 5 mM in 96-well plates incubated at 37 °C. Reaction mixtures (100 µl) containing the substrate in 100 mMTris-HCl buffer, pH 7.0, were initiated by adding 0.44 µg and 0.30 µg of enzyme Bdg42A and Bdg2A, respectively. Using these assay conditions, the optimal pH was determined with 5 mM 4-NP-β-d-galactopyranoside (pNPGal) using 100 mM phosphate-citrate buffer (pH 4.0–7.5), 100 mM Tris–HCl buffer (pH 7.5.0–8.5) and 100 mM glycine–NaOH buffer (pH 8.5.0–9.5). The optimal temperature and kinetic studies with pNPGal were performed as previously described^28^. The capability of Bdg42A and Bdg2A to hydrolyze natural oligosaccharides was assayed using several substrates listed in Tables 3 and 4. The reactions (10 µl) were performed at 37 °C for 16 h using 2 mM substrate in 100 mM Tris–HCl buffer, pH 7.0. For kinetic studies with natural oligosaccharides, varying concentrations from 1 to 15 mM substrate were used in the same buffer and the reactions were incubated at 37 °C for different periods of time ranging from 30 s to 8 min. The reaction mixtures were analyzed by chromatography using the Dionex system and column described above.

### Nucleotide sequence accession numbers

The partial nucleotide sequence of the 16S rRNA gene amplicons have been deposited at the GenBank database under the accession numbers MZ323909 to MZ323948, and the sequences of the genes encoding the β-galactosidases Bdg42A and Bdg2A from *B. dentium* strain Y510 under the accession numbers MZ313538 and MZ313539.

### Ethical approval

All applicable international, national, and/or institutional guidelines for the use of human samples were followed. The study protocol with the registration number H1544010468380 was approved by the Ethics Committee of the University of Valencia. Written informed consent was obtained from a parent and/or legal guardian.

## Supplementary Information

Supplementary Information.

## Data Availability

All data generated or analyzed during this study are included in this published article.
