# Supplementary material for: Infant-gut associated Bifidobacterium dentium strains utilize the galactose moiety and release lacto-N-triose from the human milk oligosaccharides lacto-N-tetraose and lacto-N-neotetraose
Source: Sci Rep. 2021 Dec 2;11:23328. doi: 10.1038/s41598-021-02741-x (PMC8639736; doi:10.1038/s41598-021-02741-x)

Infant-gut associated *Bifidobacterium dentium* strains utilize the galactose moiety and release lacto-*N*-triose from the human milk oligosaccharides lacto-*N*-tetraose and lacto-*N*-neotetraose

Eva M. Moya-González[1], Antonio Rubio-del-Campo[1], Jesús Rodríguez-Díaz[2] and María J. Yebra[1*].

[1]Laboratorio de Bacterias Lácticas y Probióticos, Departamento de Biotecnología de Alimentos, IATA-CSIC, Valencia, Spain. [2]Departamento de Microbiología, Facultad de Medicina, Universidad de Valencia, Valencia, Spain.

*Address correspondence to María J. Yebra, yebra@iata.csic.es

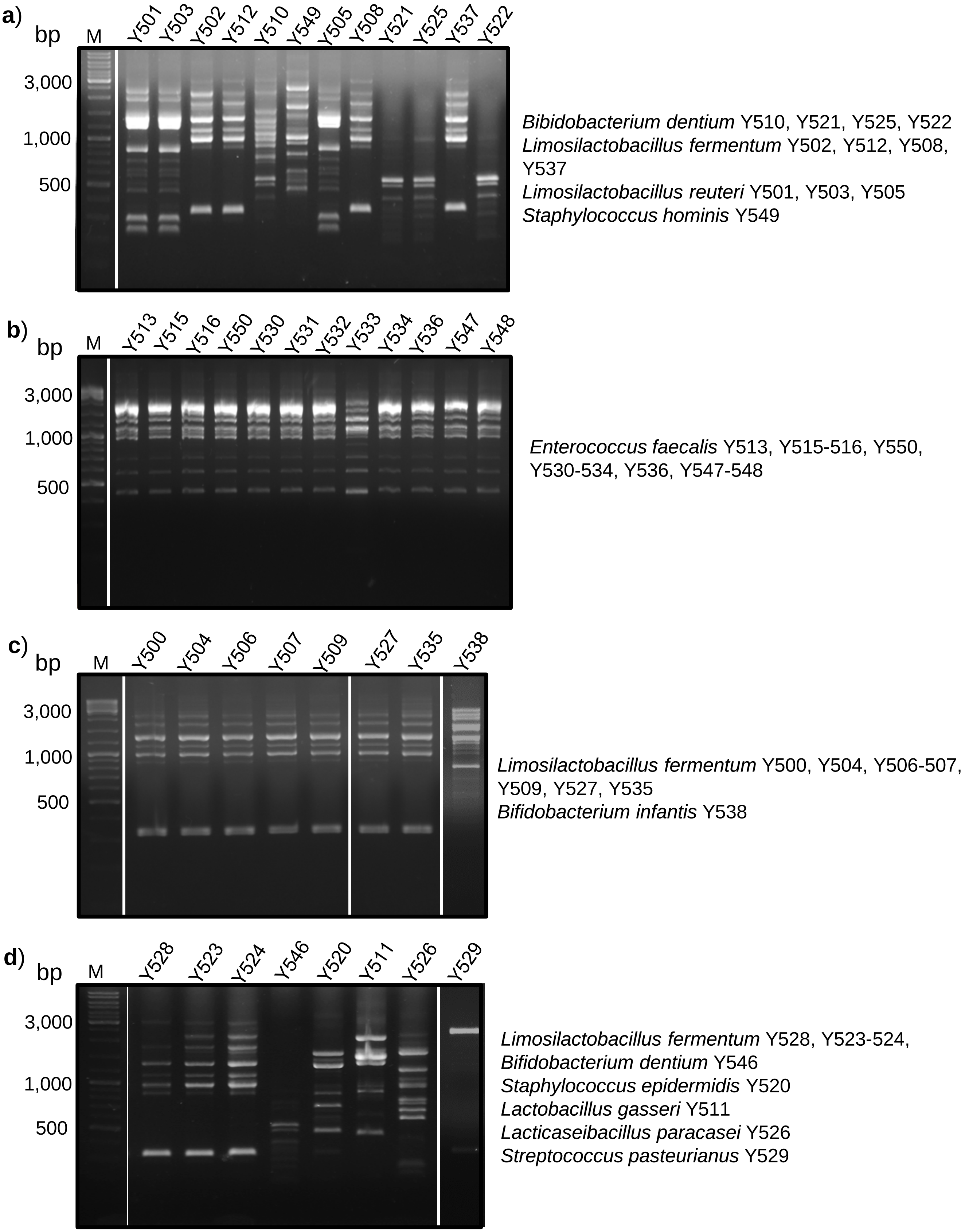

Supplementary Figure S1. Agarose gel (2%) electrophoresis (a, b, c, d) of the DNA band patterns obtained from the bacteria strains isolated in this study using RAPD-PCR analysis. Lanes in panels a and b are from the same gels, whereas lanes in panel c and d come from two different gels run under the same conditions. Lane M, DNA molecular weight marker.

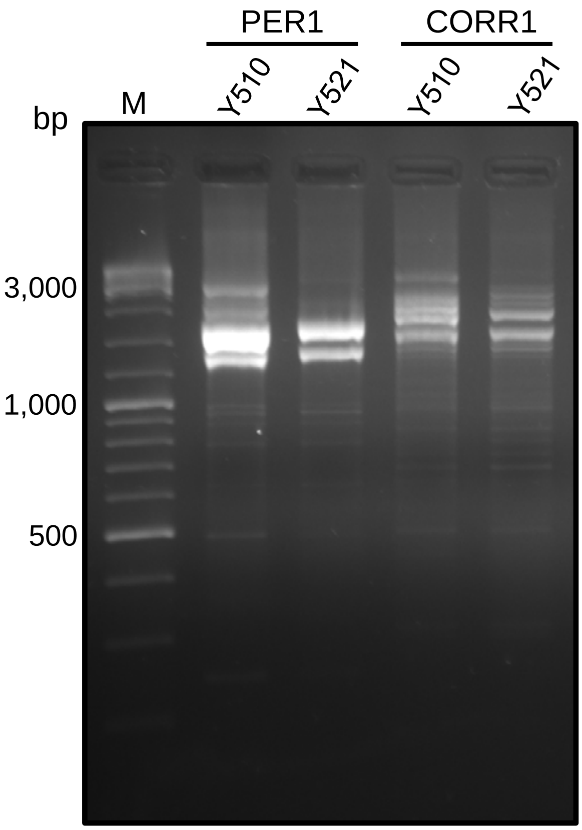

Supplementary Figure S2. Agarose gel (2%) electrophoresis of the DNA band patterns obtained from *Bibidobacterium dentium* strains Y510 and Y521 using RAPD-PCR analysis with the primers PER1 and CORR1. Lane M, DNA molecular weight marker.

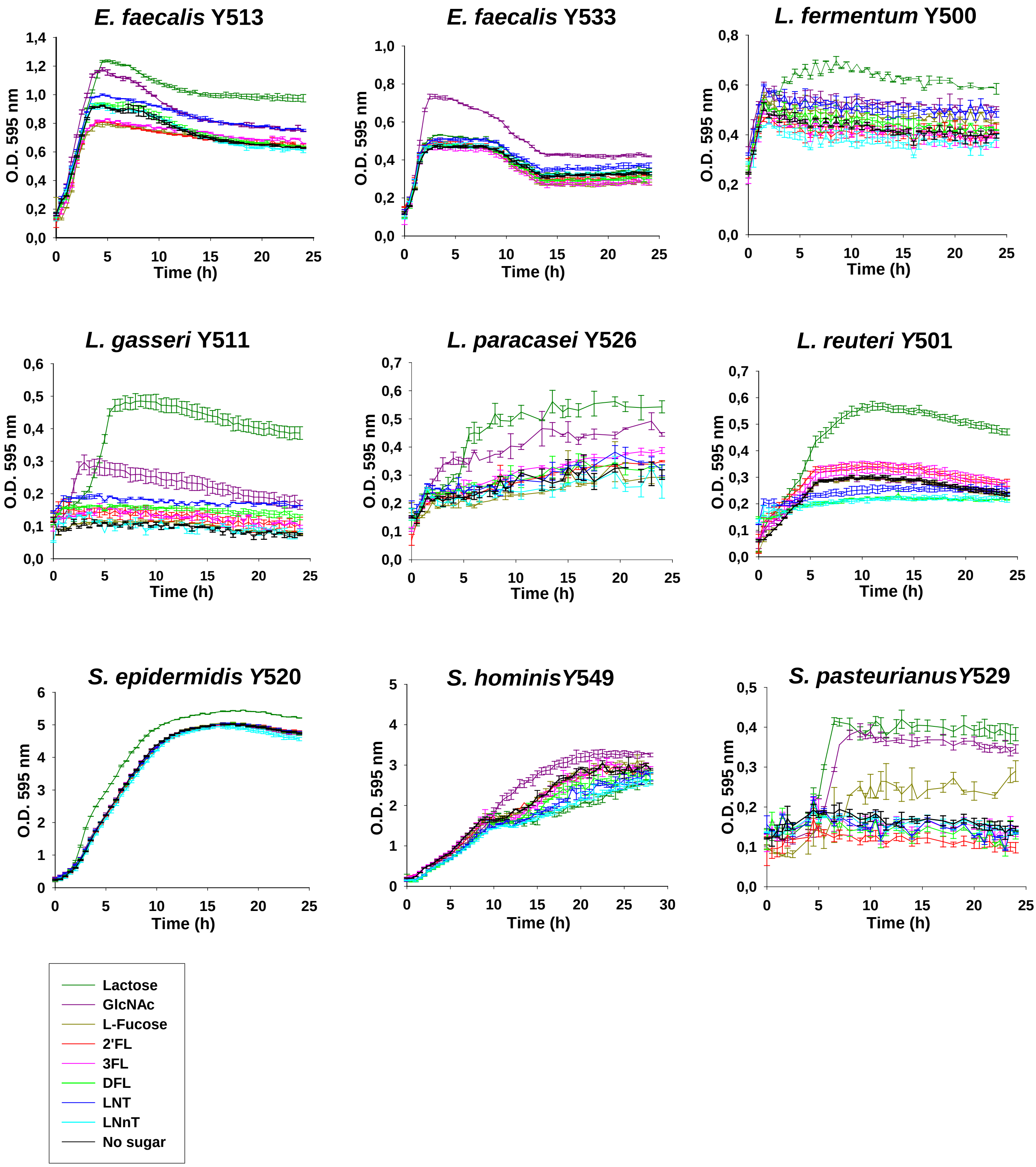

Supplementary Figure S3. Growth curves of *Enterococcus faecalis* (Y513, Y533), *Limosilactobacillus fermentum* (ex-*Lactobacillus fermentum)* (Y500), *Lactobacillus gasseri* (Y511), *Lacticaseibacillus paracasei* (ex-*Lactobacillus paracasei*) (Y526), *Limosilactobacillus reuteri* (ex-*Lactobacillus reuteri*) (Y501), *Staphylococcus epidermidis* (Y520), *Staphylococcus hominis* (Y549) and *Streptococcus pasteurianus* (Y529) on MRS basal medium without carbon source (No sugar), with 2 mM of lactose, *N*-acetylglucosamine (GlcNAc), L-fucose, 2'-fucosyllactose (2'FL), 3-fucosyllactose (3FL), difucosyllactose (DFL), lacto-*N*-tetraose (LNT) or lacto-*N*-neotetraose (LNnT).

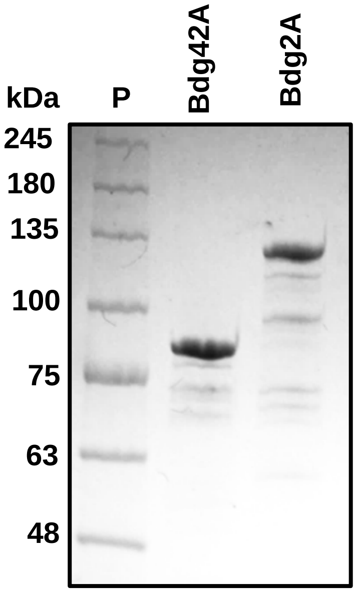

Supplementary Figure S4. Coomassie brilliant blue-stained 10% SDS-polyacrylamide gel showing the His-tagged proteins β-galactosidases Bdg42A and Bdg2A. Lane P, protein standards. The numbers on the right are molecular masses.

Supplementary_TableS1. β-Galactosidase activity in the supernatant, whole cells, permeabilized cells and crude extracts fractions of *Bifidobacterium dentium* strain Y510 cultured in MRS medium[a].

	β-galactosidase activity[b]
Supernatant (nmol/min/OD)	0.007 ± 0.006
Whole cells (nmol/min/OD)	0.242 ± 0.040
Permeabilized cells (nmol/min/OD)	1.247 ± 0.025
Cell-free crude extracts (μmol/min/mg protein)	0.241 ± 0.010

[a]β-galactosidase activity was determined with 2-nitrophenyl-β-D-galactopyranoside as the substrate.

[b]Each value represents the mean of three different measurements ± standard deviation.

Original gel of supplementary Figure_S1 a)

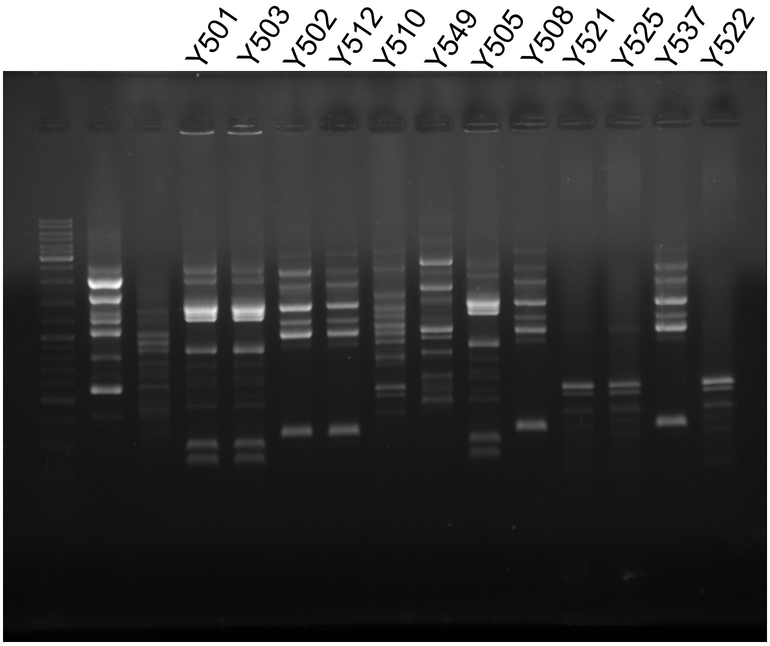

Original gel of supplementary Figure_S1 b)

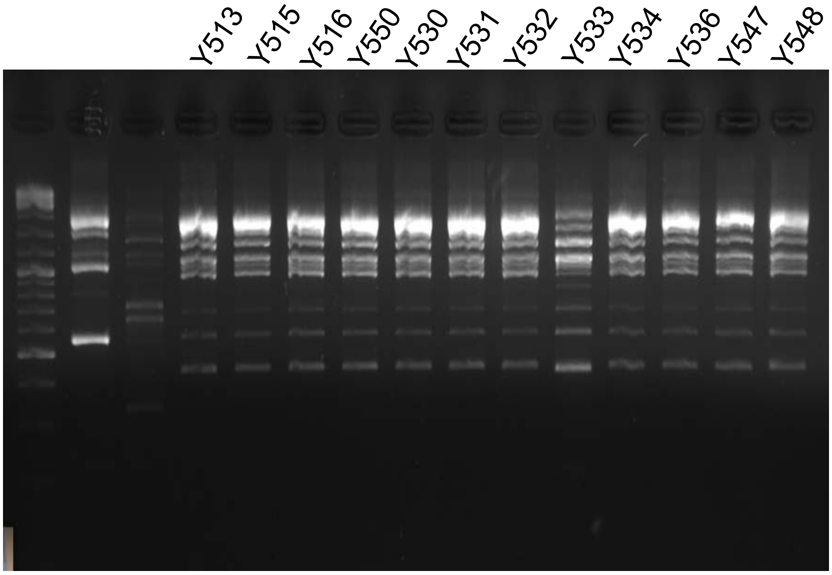

Original gels of supplementary Figure_S1 c)

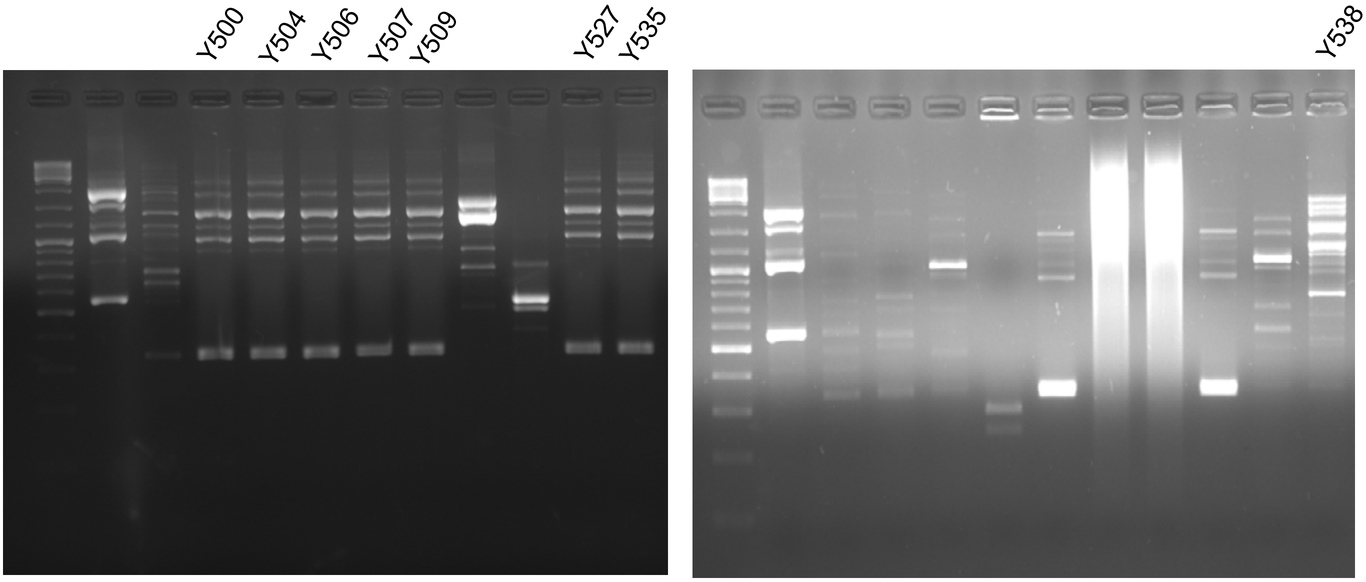

Original gels of supplementary Figure_S1 d)

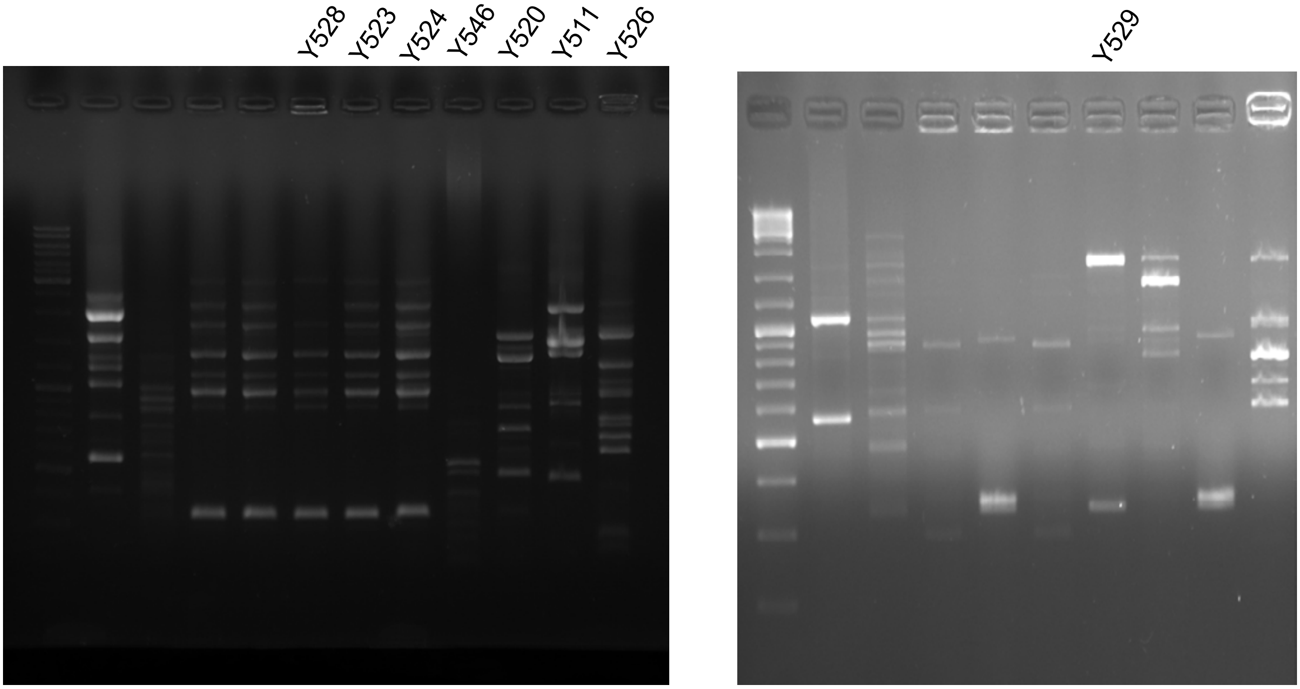

Supplement: Supplementary file 1 — Supplementary Information. [file 41598_2021_2741_MOESM1_ESM.pdf]
